# Role of α‐SMA, NOX4, Fibronectin, and HIF‐1α in Pulmonary Microvascular Remodeling in COPD Patients Without Pulmonary Arterial Hypertension

**DOI:** 10.1111/crj.70218

**Published:** 2026-08-07

**Authors:** Guo Xiaotong, Fan Yuchun, Ji Ting, Cao Xia, Jiang Haifeng, Chen Juan, Xin Hongxia

**Affiliations:** ^1^ Department of Respiratory and Critical Care Medicine The General Hospital of Ningxia Medical University Yinchuan China; ^2^ Department of Pathology The General Hospital of Ningxia Medical University Yinchuan China

**Keywords:** chronic obstructive pulmonary disease, extracellular matrix, HIF‐1α, NOX4, pulmonary microvessels, pulmonary vascular remodeling

## Abstract

**Background:**

Pulmonary arterial hypertension (PAH) is a severe chronic obstructive pulmonary disease (COPD) complication, with vascular remodeling occurring early in disease progression. Oxidative stress, particularly NADPH oxidase subunit 4 (NOX4) activity, drives vascular smooth muscle differentiation, vasoconstriction, and proliferation, while Hypoxia‐inducible factor‐1α (HIF‐1α), activated by NOX4‐derived hydrogen peroxide (H_2_O_2_), plays a key contributory role in early pulmonary vascular remodeling. This study explores the roles of α‐smooth muscle actin (α‐SMA), NOX4, fibronectin (FN), and HIF‐1α roles in pulmonary microvascular remodeling in COPD patients without PAH.

**Methods:**

Lung tissue samples from patients undergoing resection at General Hospital of Ningxia Medical University (Oct 2022–Dec 2023) were analyzed. Participants included COPD (*n* = 20) and control (*n* = 17) groups. NOX4, HIF‐1α, α‐SMA, and fibronectin expression in the pulmonary microvasculature (< 500 μm) were assessed via immunohistochemistry and Western blot. Smooth muscle remodeling (pulmonary vascular smooth muscle thickness percentage, WT%; pulmonary vascular smooth muscle area percentage, WA%) and serum vascular endothelial growth factor (VEGF) levels were evaluated.

**Results:**

COPD patients exhibited significant thickening of the pulmonary microvascular smooth muscle layer, with endothelial cell swelling, degeneration, and necrosis. The WT% and WA% values of COPD patients were elevated compared to controls, alongside increased FN expression in the pulmonary microvasculature. NOX4, HIF‐1α, and α‐SMA expression levels were higher in COPD patients, particularly within endothelial cells and macrophages surrounding pulmonary vessels. The peripheral blood neutrophil count in the COPD group was higher than in the control group. Compared to the control group, COPD patients showed high expression of NOX4 and HIF‐1α in macrophages surrounding the pulmonary microvessels. Serum VEGF concentrations were significantly elevated in COPD patients and positively correlated with HIF‐1α expression in lung tissue homogenates.

**Conclusions:**

Pulmonary microvascular remodeling occurs in COPD patients even without PAH, characterized by smooth muscle thickening, extracellular matrix deposition, and endothelial alterations. The NOX4‐HIF‐1α oxidative stress pathway, coupled with inflammatory cell activation, may contribute to microvascular remodeling in COPD.

## Graphical abstract

This study investigates pulmonary microvascular remodeling in non‐hypertensive COPD patients, focusing on α‐SMA, NOX4, fibronectin, and HIF‐1α. The findings reveal key roles of these proteins and serum VEGF in vascular changes, offering new insights into COPD pathogenesis.

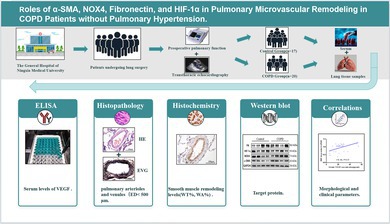

## 1 Introduction

Chronic obstructive pulmonary disease (COPD) ranks as the fourth leading cause of death worldwide [1]. The pathogenesis of pulmonary vascular remodeling in COPD is complex and can be triggered by multiple factors, including chronic hypoxia, inflammation, and smoking. Pulmonary vascular remodeling within the pulmonary circulation impairs gas exchange in the alveolar space, leading to the development of pulmonary arterial hypertension (PAH) in COPD patients. Although PAH is typically diagnosed in the advanced stages of COPD, pulmonary vascular remodeling begins early in the disease course [2, 3]. The primary characteristics of vascular remodeling in COPD include smooth muscle cell proliferation, endothelial dysfunction, and extracellular matrix (ECM) protein deposition [4, 5]. While numerous studies have indicated that pulmonary vascular remodeling in COPD predominantly occurs in the pulmonary arterioles, research focusing on the mechanisms underlying remodeling of pulmonary venules remains scarce. Addressing this gap is essential for a comprehensive understanding of COPD‐associated vascular pathology.

The overproduction of reactive oxygen species (ROS) plays a significant role in pulmonary vascular remodeling. They are primarily generated by mitochondria and serves as critical intracellular and intercellular messengers that promote pulmonary vascular smooth muscle cell proliferation and inhibit apoptosis. The NADPH oxidase (NOX) family is the only known enzymatic system responsible for the primary production of ROS. Among the NOX isoforms, NADPH oxidase subunit 4 (NOX4) is a major player in hypoxia‐induced pulmonary circulatory disturbances. Its activation leads to increased hydrogen peroxide (H_2_O_2_) levels in vascular smooth muscle cells, mediating their proliferation and migration, thereby contributing to the development of PAH [6]. Under hypoxic conditions, pulmonary artery endothelial cells release ROS, which subsequently diffuse to surrounding endothelial cells, triggering calcium influx and leading to pulmonary vessel constriction [7]. Hypoxia‐inducible factor‐1α (HIF‐1α) is a key oxygen sensor that plays a crucial role in vascular remodeling, PAH, fibrosis, and common comorbidities of COPD [8]. NOX4 is a target gene of HIF‐1α under hypoxic conditions, where HIF‐1α binds to the hypoxia response element of the NOX4 promoter, enhancing its activity. This process aids in the regulation of ROS levels following hypoxic exposure and promotes pulmonary vascular smooth muscle cell proliferation. Despite these findings, the specific roles of NOX4 and HIF‐1α in pulmonary venular remodeling remain unclear. We hypothesized that activation of the NOX4–HIF‐1α axis is associated with early microvascular remodeling. Investigating their involvement in COPD patients without PAH could provide novel insights into early pulmonary microvascular alterations and potential therapeutic strategies.

Additionally, inflammation plays a central role in COPD progression, with increased macrophage, neutrophil, and lymphocyte infiltration contributing to emphysema and vascular remodeling [9]. Compared to healthy individuals, COPD patients show a marked increase in the number of activated inflammatory cells in the lungs, which release higher levels of superoxide anion radicals (O_2_^−^) and H_2_O_2_. Inflammation also plays a critical role in the pathogenesis of PAH, with studies indicating the accumulation of a large number of inflammatory cells, including macrophages, lymphocytes, and mast cells, around the blood vessels in PAH patients [10]. In macrophages, the role of NOX is to generate the “oxidative burst,” aimed at killing and destroying engulfed pathogens. Thus, NOX4 participates in the hypoxic and inflammatory responses in COPD pulmonary vasculature and may serve as a novel therapeutic target for PAH treatment.

Building upon our previous findings regarding NOX4 in distal arterioles [11], the pulmonary veins are highly sensitive to hypoxia, similar to the pulmonary arteries. Under hypoxic conditions, pulmonary veins like pulmonary arteries will experience smooth muscle proliferation and migration, vasoconstriction, and vascular structural remodeling, mainly located in the distal pulmonary veins [12, 13]. The present study extends this investigation to pulmonary venules and incorporates fibronectin (FN) and vascular endothelial growth factor (VEGF) to provide a comprehensive view of remodeling in COPD patients without diagnosed PAH. This study aims to investigate the expression levels and roles of different proteins in the remodeling of pulmonary arterioles and venules in COPD patients without PAH. By addressing these gaps, this research may provide valuable insights into early vascular alterations in COPD and pave the way for future therapeutic interventions targeting oxidative stress and hypoxia‐related pathways.

## 2 Methods

This study adhered to the ethical guidelines approved by the Ethics Committee of the General Hospital of Ningxia Medical University (KYLL‐2023‐0422). This study was conducted in accordance with the Declaration of Helsinki (as revised in 2024). All participants provided informed consent for the use of clinical data in scientific research during hospitalization and signed written consent for the publication of collected data.

**FIGURE 1:**
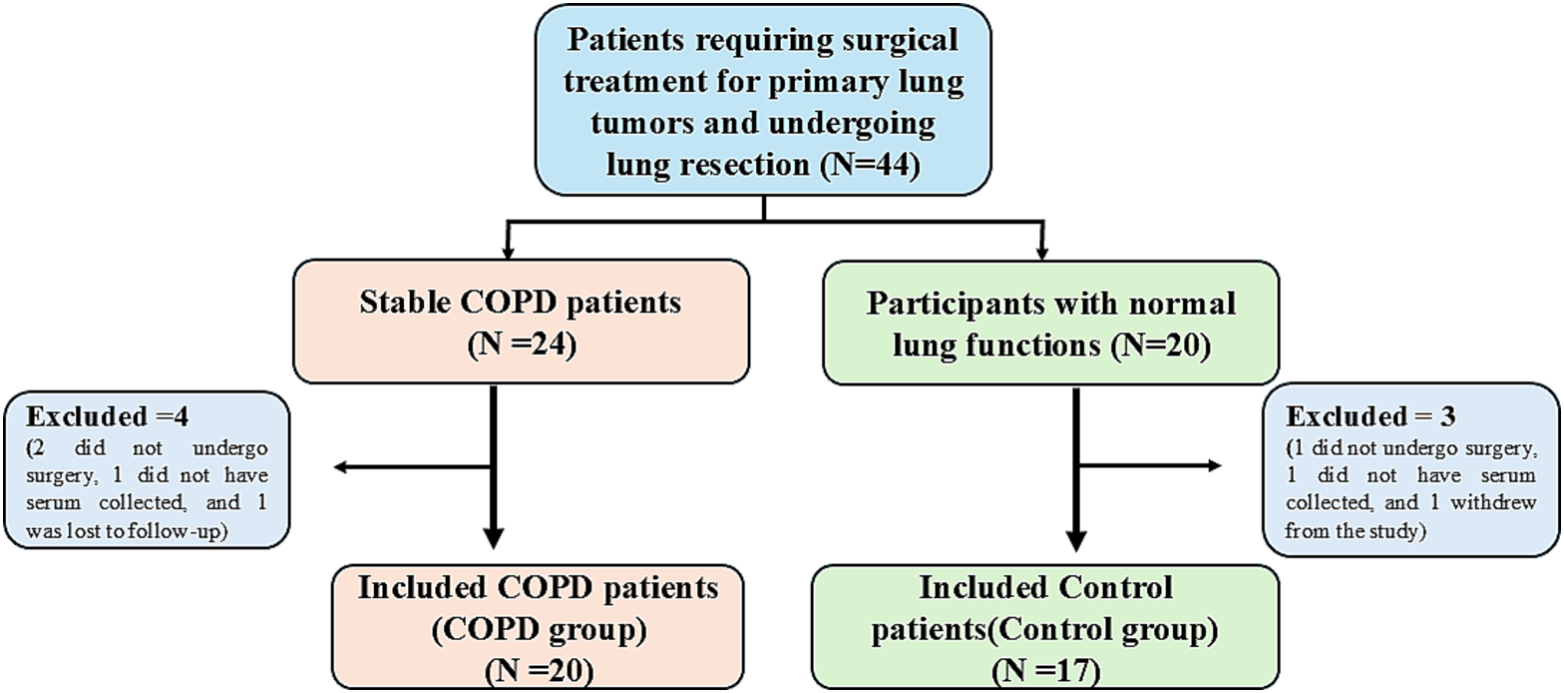
Flowchart of participants in the cohort study. Abbreviations: COPD, chronic obstructive pulmonary disease.

The diagnostic criteria for COPD followed GOLD 2022 guidelines [14]. Participants were categorized into two groups based on their preoperative pulmonary function test results (Figure 1): COPD Group: 20 COPD patients were selected out of 24, with 4 exclusions (2 did not undergo surgery, 1 lacked serum collection, and 1 was lost to follow‐up). This group included 13 patients with GOLD I severity and 7 with GOLD II severity. Control Group: 17 control patients with normal lung function, exhibiting no signs of obstructive airway disease, were chosen out of 20, with 3 exclusions (1 did not undergo surgery, 1 lacked serum collection, and 1 withdrew from the study).

All participants underwent transthoracic echocardiography to measure peak tricuspid regurgitation velocity during systole, ensuring the exclusion of patients with PAH. Detailed characteristics of the entire patient population have been described elsewhere [11]. The transthoracic echocardiography measurement method and the calculation of each parameter mainly refer to the adult echocardiography assessment and examination measurement guidelines. The main parameters include the estimated systolic pulmonary artery pressure (SPAP) and right atrial pressure (RAP). SPAP can be estimated by measuring the peak tricuspid regurgitation velocity (TRV), which is, calculated according to the simplified Bernoulli equation [SPAP = 4 (V) [2] + RAP] (V is TRV, calculated in m/s). SPAP > 40 mmHg is defined as pulmonary hypertension [15].

### 2.1 Tissue Sample Collection

To ensure the integrity of the study population, serum and lung tissue samples were collected during surgical procedures. Lung tissue was obtained from areas distant from the tumor, and pathological examination confirmed the absence of obstructive pneumonia or tumor invasion.

### 2.2 Histological Analysis of Pulmonary Vasculature

3–4 μm (μm) thick sections were stained with Hematoxylin and eosin (HE, LeiGen Company, China) staining, along with Elastic Fiber (ELASTIC) Verhoeff‐Van Gieson Staining Elastic Fiber (ELASTIC) Verhoeff‐Van Gieson Staining [16] (EVG, GENMED Scientifics Inc., USA) elastic fiber staining, which were utilized to identify pulmonary arterioles and venules.

### 2.3 Vascular Morphological Analysis

From each lung tissue section, 3–5 structurally intact small vessels (pulmonary arterioles and venules, external diameter < 500 μm) were randomly selected for high‐magnification observation. Measurements included external diameter (ED), internal diameter, smooth muscle area, and total vascular area. Pulmonary vascular remodeling was evaluated by calculating the percentage of smooth muscle thickness (WT%) and smooth muscle area (WA%) using the following formulas: WT% = (vascular ED—vascular internal diameter)/vascular ED × 100, WA% = smooth muscle area / total vascular area × 100 [17]. For vascular smooth muscle cell identification and assessment of proliferative smooth muscle formation, α‐smooth muscle actin (α‐SMA) immunostaining was performed using α‐SMA antibodies.

### 2.4 Immunohistochemistry (IHC)

Lung tissue was sectioned into 3–4 μm paraffin slices for histological analysis. The sections were incubated with primary antibodies against α‐SMA (Abcam, MA, USA), NOX4 (Novus Biotech, CO, USA), HIF‐1α (Abcam, MA, USA), and FN (Abcam, MA, USA), each at a 1:500 dilution.3–5 small vessels (pulmonary arterioles and venules, external diameter < 500 μm) per subject were evaluated for the average optical density (AOD) of target protein in lung tissue. Five randomly selected fields of each section at a magnification of ×400 were used for analyzing the positive staining as previously reported [11] The obtained images were then used for a semi‐quantitative analysis of the expression of the protein of interest by measuring the integrated absorbance (IA) or optical density (OD) using the Image‐Pro Plus 6.0 (IPP) software (Media Cybernetics, MD, USA), and the AOD values of each sample were used as an index of the expression of proteins.

### 2.5 Image Analysis

To measure the vascular smooth muscle area, Image‐Pro Plus 6.0 (IPP) image analysis software was utilized. Using the obtained data, the following parameters were calculated [11]:

vascular wall area (WA): Defined as the area of the smooth muscle layer, calculated as WA = TA (the total area of blood vessels) − LA (luminal area of blood vessels), wall thickness (WT): determined as WT =  WA/P (perimeter of blood vessels), vessel radius (*R*): Calculated as *R* = P/2π. External Diameter (ED): Defined as ED = *R* + WT/2. Calculated as

$$\mathrm{WT}\%=\left(\times \mathrm{WT}\right)/\mathrm{ED}\times 100.\text{Calculated}\;\mathrm{as}\;\mathrm{WA}\%=\mathrm{WA}/\mathrm{TA}\times 100.$$

### 2.6 Western Blot Analysis

Western blot analysis was performed following standard protocols using the Bio‐Rad 132 system. Primary antibodies were applied at the following dilutions: α‐SMA (1:2000), NOX4 (1:2000), HIF‐1α (1:2000), FN (1:2000), and GAPDH (1:2000, Abcam, MA, USA). Protein expression levels were normalized to the GAPDH internal control, and the results are presented as fold changes relative to the control group.

### 2.7 Enzyme‐Linked Immunosorbent Assay (ELISA)

Serum levels of VEGF were quantified using the Human VEGF ELISA Kit (JL18341) in accordance with the manufacturer's protocol. Absorbance at 450 nm was recorded using a microplate reader (Thermo Fisher Scientific, Finland).

### 2.8 Statistical Analysis

Quantitative data are expressed as the mean ± standard deviation [SD]. Group comparisons were conducted using an independent samples *t*‐test for continuous variables. Normality of data distribution was assessed using the Shapiro–Wilk test. For ordinal data, either the Chi‐square test was used. Correlations between morphological and clinical parameters were analyzed using Pearson's correlation coefficient. Statistical significance was defined as a *p*‐value < 0.05.

## 3 Results

### 3.1 Clinical Data

There were no notable differences in age, gender distribution, and smoking index between the control and COPD groups, indicating comparable baseline characteristics. However, the peripheral blood neutrophil count was significantly elevated in the COPD group compared to the control group (*p* < 0.001), reflecting heightened inflammatory activity in COPD patients (Table 1). Moreover, both the FEV_1_/FVC ratio and the percentage of the predicted value for FEV_1_ (FEV_1_% pred) were markedly reduced in the COPD group compared to the control group (*p* < 0.001 and *p* = 0.04, respectively), underscoring the impaired lung function associated with COPD.

**TABLE 1:** General clinical characteristics.

Item	Control (*n* = 17)	COPD (*n* = 20)	*p*
Age (years)	56.0 ± 7.42	58.8 ± 5.84	0.22
Male/Female (n)	11/6	12/8	1.00 (*χ* ^2^ = 1.33)
Smoking index (pack‐years)	9.92 ± 13.54	18.5 ± 20.3	0.15
FEV_1_/FVC (%)	76.9 ± 5.58	62.4 ± 7.46	**< 0.001**
FEV_1_% (predicted %)	98.9 ± 20.4	85.1 ± 19.2	**0.04**
Peripheral blood neutrophil count (×10^9^/L)	2.80 ± 0.65	4.03 ± 1.56	**< 0.001**

*Note:* Bold values indicate statistical significance (*p* < 0.05).

Abbreviations: COPD, chronic obstructive pulmonary disease; FEV1/FVC, forced expiratory volume in one second/forced vital capacity; FEV1% pred, forced expiratory volume in one second total predicted value.

### 3.2 Morphology and Degree Remodeling of Pulmonary Microvessels

**FIGURE 2:**
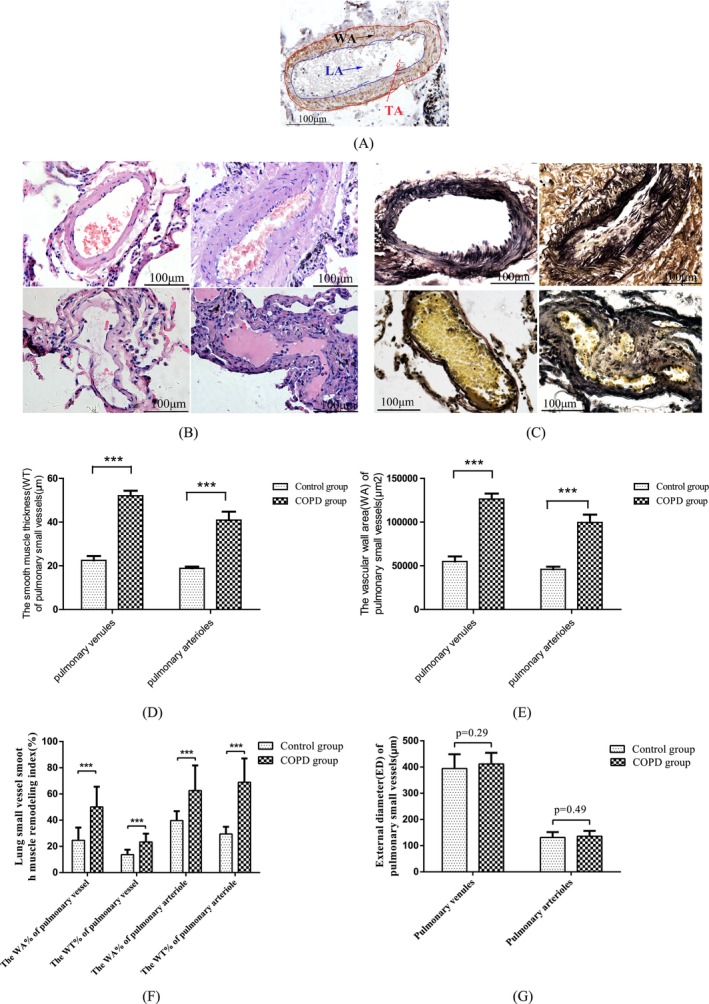
Pathomorphological assessment of pulmonary vascular remodeling. **A** The software was employed to quantify the total area of blood vessels (TA, outlined by the red line), the luminal area (LA, outlined by the blue line), Vascular Wall Area (WA) = TA—LA, the region between the red and blue lines. **B** Pulmonary microvessels hematoxylin and eosin staining (HE) Staining (×400) **C** Pulmonary microvessels elastic fiber (ELASTIC) Verhoeff‐Van Gieson (EVG) staining (×400). **D** The comparison of smooth muscle thickness (WT) of pulmonary small vessels (μm), **E** Vascular wall area (WA) of pulmonary small vessels (μm^2^). **F** lung small vessels smooth muscle remodelling index (%) in pulmonary vessels and pulmonary arteries. **G** External diameter (ED) of pulmonary vessels and pulmonary arteries. Abbreviations: WT%, pulmonary vascular smooth muscle thickness percentage; WA%, pulmonary vascular smooth muscle area percentage. Compared to control group, *p < 0.05, *** *p* < 0.01 (*N* = 17 for control group; *N* = 20 for COPD group).

The (Figure 2A) illustrates the method of measuring vascular smooth muscle area. The HE (Figure 2B) and EVG (Figure 2C) stains were employed to examine lung tissue structure and identify pulmonary arterioles and venules. The pulmonary microvessels were observed to have well‐defined boundaries and intact structures in the control group compared to COPD group. Pulmonary venules displayed distinct endothelial linings, a thin monolayer of elastic fibers, and identifiable smooth muscle cells. Additionally, the endothelial and smooth muscle cells of pulmonary arterioles were arranged in an orderly manner. In contrast, the COPD group showed disrupted alveolar architecture with thickened alveolar septa. The boundaries of pulmonary microvessels were blurred and damaged, accompanied by significant inflammatory cell infiltration surrounding the vessels. Pulmonary venules exhibited increased wall thickness, with fibrous elastic deposits in the intima and media layers, mild proliferation of myofibroblasts, and noticeable collagen and elastin accumulation. Furthermore, endothelial cells in pulmonary arterioles were swollen, degenerated, and necrotic, while smooth muscle cells demonstrated proliferation. This led to uneven thickening and distortion of the vessel wall, resulting in narrowed lumens (Figure 2B,C).

The smooth muscle thickness (Figure 2D) and vascular wall area (Figure 2E) of pulmonary small vessels were significantly higher in the COPD group. Thus, the WA% and WT% in the pulmonary venules and arterioles of the COPD group were significantly (*p* < 0.01) higher than those in the control group (Figure 2F). However, there were no significant differences (*p* > 0.05) in the ED of pulmonary venules and arterioles between the COPD group and the control group (Figure 2G and Table 2).

**TABLE 2:** Degree remodeling of pulmonary microvessels.

	Control (*n* = 17)	COPD (*n* = 20)	*p*
The ED of pulmonary venules (μm)	394.94 ± 54.37	411.84 ± 42.54	0.29
The ED of pulmonary arterioles (μm)	131.29 ± 20.44	136.02 ± 20.62	0.49
Vascular wall area (WA) of pulmonary venules (μm2)	54954.74 ± 23381.33	126391.34 ± 28064.79	**< 0.01**
Vascular wall area (WA) of pulmonary arterioles (μm2)	45962.36 ± 12037.77	99609.16 ± 40003.86	**< 0.01**
The comparison of smooth muscle thickness (WT) of pulmonary venules (μm)	22.48 ± 8.24	52.09 ± 10.21	**< 0.01**
The comparison of smooth muscle thickness (WT) of pulmonary arterioles (μm)	18.81 ± 3.41	40.91 ± 17.35	**< 0.01**
The WA% of pulmonary venules (%)	24.63 ± 9.78	50.14 ± 15.37	**< 0.01**
The WA% of pulmonary arterioles (%)	39.69 ± 7.18	62.65 ± 19.12	**< 0.01**
The WT% of pulmonary venules (%)	13.72 ± 3.72	23.36 ± 6.36	**< 0.01**
The WT% of pulmonary arterioles (%)	29.45 ± 5.52	68.93 ± 18.23	**< 0.01**

*Note:* Bold values indicate statistical significance (*p* < 0.05).

Abbreviations: COPD, chronic obstructive pulmonary disease; ED, external diameter; WA, vascular wall area; WA%, pulmonary vascular smooth muscle area percentage; WT, wall thickness; WT%, pulmonary vascular smooth muscle thickness percentage.

### 3.3 Expression of α‐SMA and NOX4in Pulmonary Microvessels

**FIGURE 3:**
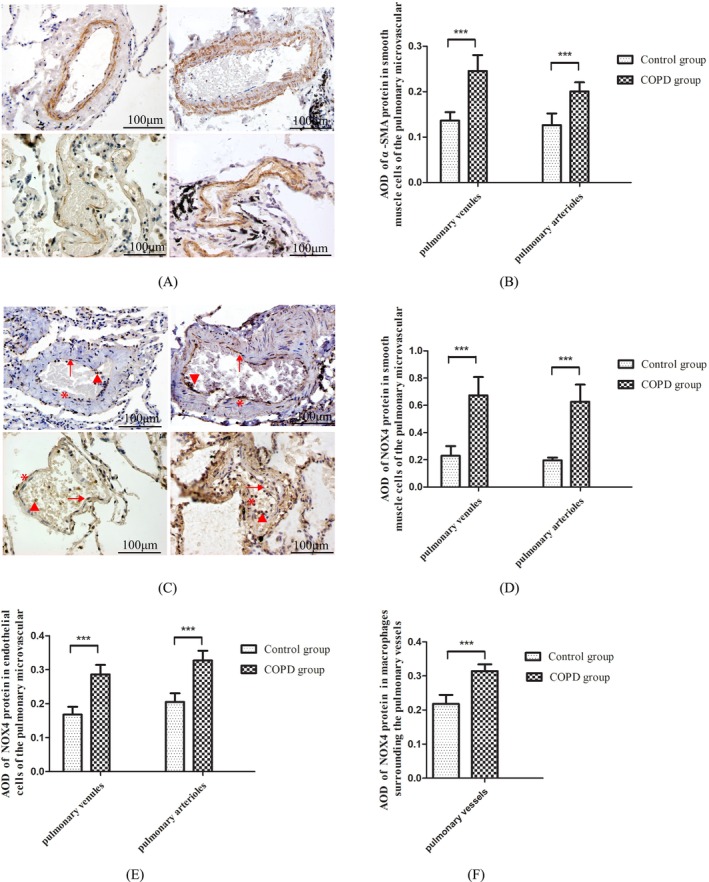
Protein expression of α‐SMA and NOX4 in lung tissue. **A** Representative images of pulmonary microvascular smooth muscle cell α‐SMA immunohistochemical staining (IHC × 400). **B** Immunohistochemical detection of α‐SMA protein expression in pulmonary microvascular smooth muscle cells. **C** Representative images of NOX4 expression in pulmonary microvascular smooth muscle cells, endothelial cells, and macrophages surrounding pulmonary vessels (IHC × 400). **D** Immunohistochemical detection of NOX4 protein expression in pulmonary microvascular smooth muscle cells. **E** Immunohistochemical detection of NOX4 protein expression in pulmonary microvascular endothelial cells. **F** Immunohistochemical detection of NOX4 protein expression in macrophages surrounding the pulmonary vessel. Arrowheads represent endothelial cells, stars represent smooth muscle cells, and triangles represent macrophages. Abbreviations: COPD, chronic obstructive pulmonary disease; α‐SMA, α‐smooth muscle actin; NOX4, NADPH oxidase subunit 4; AOD, average optical density. Compared to control group, *p < 0.05, *** *p* < 0.01 (*N* = 17 for control group; *N* = 20 for COPD group).

The expression of α‐SMA protein in smooth muscle cells of the pulmonary venules and arterioles determined by AOD values of IHC was higher in the COPD group compared to the control group (*p* < 0.01) (Figure 3A,B). The expression of NOX4 protein in smooth muscle cells and endothelial cells of the pulmonary venules and arterioles determined by AOD values of IHC in the COPD group was significantly higher than that in the control group (*p* < 0.01) (Figure 3C–E).

The expression of NOX4 protein in macrophages surrounding the pulmonary vessels determined by AOD values of IHC in the COPD group was also significantly increased compared to the control group (*p* < 0.01) (Figure 3F).

### 3.4 Expression of HIF‐1α and FNproteins in Pulmonary Microvessels

**FIGURE 4:**
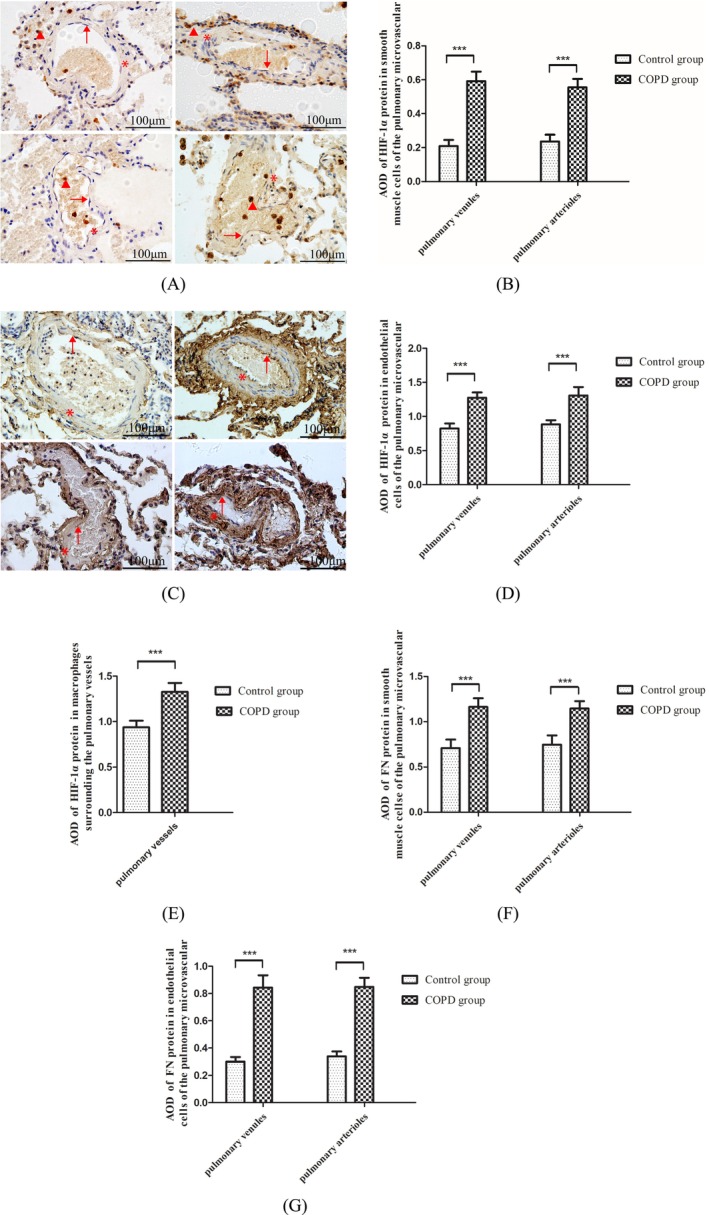
Protein Expression of HIF‐1α and FN in Lung Tissue. **A** Representative images of HIF‐1α expression in pulmonary microvascular smooth muscle cells, endothelial cells, and macrophages (IHC × 400), **B** Immunohistochemical detection of HIF‐1α protein expression in pulmonary microvascular smooth muscle cells. **C** Representative images of FN expression in pulmonary microvascular smooth muscle cells and endothelial cells (IHC × 400). **D** Immunohistochemical detection of HIF‐1α protein expression in pulmonary microvascular endothelial cells. **E** Immunohistochemical detection of HIF‐1α protein expression in macrophages surrounding the pulmonary vessel. **F** Immunohistochemical detection of FN protein expression in pulmonary microvascular smooth muscle cells. **G** Immunohistochemical detection of FN protein expression in pulmonary microvascular endothelial cells. Arrowheads represent endothelial cells, stars represent smooth muscle cells, and triangles represent macrophages. Abbreviations: COPD, chronic obstructive pulmonary disease; HIF‐1α, Hypoxia‐inducible factor‐1α; FN, fibronectin; AOD, average optical density. Compared to control group.*p < 0.05, *** *p* < 0.01 (*N* = 17 for control group; *N* = 20 for COPD group).

The expression of HIF‐1α protein in smooth muscle cells and endothelial cells of the pulmonary venules and arterioles determined by AOD values of IHC in the COPD group was significantly higher than that in the control group (*p* < 0.01) (Figure 4A,B,D). The expression of HIF‐1α protein in macrophages surrounding the pulmonary vessels determined by AOD values of IHC in the COPD group was also significantly increased compared to the control group (*p* < 0.01) (Figure 4E). Similarly, the expression of FN protein in smooth muscle cells and endothelial cells of the pulmonary venules and arterioles determined by AOD values of IHC in the COPD group was significantly higher than that in the control group (*p* < 0.01) (Figure 4C,F,G).

### 3.5 Western Blot Analysis and Correction With VEGF Concentrations

**FIGURE 5:**
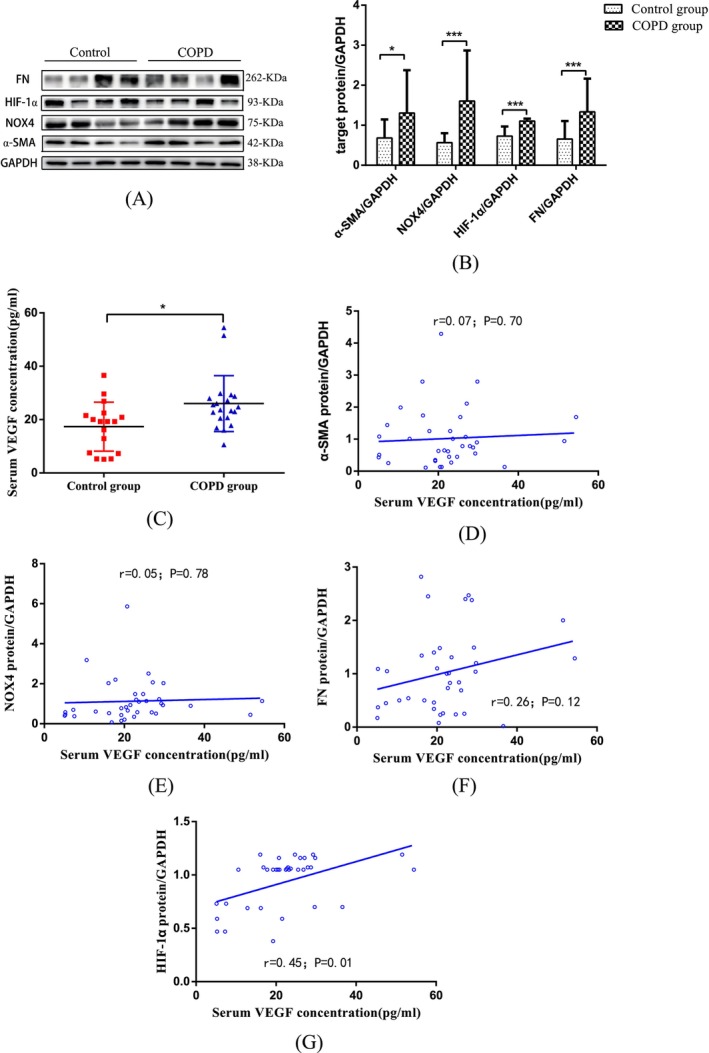
Expression of key proteins and correlation analysis with serum VEGF.**A** Western blotting analysis of target proteins in lung tissue. **B** Comparison graphs between the expression of proteins in control and COPD groups. **C** Serum VEGF detection in control and COPD groups (ELISA method), **D–G** Correlation between serum VEGF concentration and α‐SMA, NOX4, FN, and HIF‐1α protein expressions. Abbreviations: COPD, chronic obstructive pulmonary disease; FN, fibronectin. Compared to control group; HIF‐1α, Hypoxia‐inducible factor‐1α; NOX4, NADPH oxidase subunit 4; α‐SMA, α‐smooth muscle actin. VEGF: vascular endothelial growth factor; ELISA, Enzyme‐Linked Immunosorbent Assay, Compared to control group, *p < 0.05, *** *p* < 0.01 (*N* = 17 for control group; *N* = 20 for COPD group).

To verify the results of IHC staining, Western blotting was used to quantify the total expression of target proteins in whole lung tissue homogenates from all subjects. The results showed that the expression levels of α‐SMA/GAPDH, NOX4/GAPDH, HIF‐1α/GAPDH, and FN/GAPDH proteins in the COPD group were significantly higher than those in the control group (*p* < 0.05) (Figure 5A,B). The serum VEGF concentration in the COPD group (26.01 ± 10.46 pg/mL) was significantly higher than that in the control group (17.36 ± 9.18 pg/mL) (*p* < 0.05, Figure 5C). There was no correlation found between serum VEGF concentration and α‐SMA (Figure 5D), NOX4 (Figure 5E), and FN (Figure 5F) protein expressions in lung tissue homogenates (*r* = 0.05, *p* = 0.78). However, a positive correlation was observed between serum VEGF concentration and HIF‐1α protein expression (Figure 5G) in lung tissue homogenates in all participants (*r* = 0.45, *p* < 0.05).

## 4 Discussion

The study focused on the COPD group with no symptoms of PAH for exploring α‐SMA, NOX4, FN, and HIF‐1α roles in pulmonary microvascular remodeling in COPD patients without PAH. The results demonstrated that both the FEV_1_/FVC ratio and FEV_1_% predicted values were reduced in the COPD group. These findings align with existing literature, where the FEV_1_/FVC ratio is commonly used as a diagnostic criterion for COPD and is recognized as being significantly associated with increased mortality [18]. Similarly, absolute neutrophil count is strongly related to long‐term mortality in COPD and to the frequency of exacerbations and hospitalizations [19]. Our study shows an increase in the peripheral blood neutrophil count in the COPD group, reflecting systemic inflammation.

The study revealed the higher expression of α‐SMA protein in smooth muscle cells of both the pulmonary arterioles and venules of the COPD group compared to the control group. Additionally, remodeling indices of the smooth muscle layer, such as WT% and WA%, were elevated in the pulmonary arterioles and venules in the COPD group. The HE and EVG stains revealed marked swelling, degeneration, and necrosis of endothelial cells within the pulmonary arterioles and venules of the COPD group. The pathophysiological links between vascular diseases and COPD include PAH, hypoxia, systemic inflammation, and oxidative stress [20]. A unique feature of the pulmonary circulation is its ability to constrict in response to hypoxia, and in chronic, widespread hypoxia, pulmonary vascular remodeling occurs. The study by Green DE et al. [21] showed that chronic hypoxia leads to vascular remodeling in small pulmonary vessels (less than 100 μm) in mice, with thickening of the vessel walls and an increase in the number of α‐SMA‐positive vessels. Pulmonary vascular remodeling and endothelial dysfunction also occur in mild COPD patients without hypoxemia [4]. Dysfunction of pulmonary artery endothelial cells contributes to PAH development by promoting the migration and proliferation of pulmonary artery smooth muscle cells. Khushboo Goel et al. [1] found that in the absence of evidence of PAH hemodynamics, significant morphological and endothelial cell changes occurred in the small pulmonary arteries and microvessels of COPD lungs, suggesting that significant remodeling of the pulmonary small vessel and microvascular bed occurred before or independently of the clinical development of PH.

In COPD patients, elevated expression levels of the ECM protein, FN, are observed in both smooth muscle cells and endothelial cells within the pulmonary arterioles and venules compared to the control group. This heightened ECM presence suggests an ongoing process of pulmonary vascular remodeling, which includes smooth muscle thickening, an increase in vascular area, endothelial cell alterations, and ECM deposition. Notably, these changes occur before the onset of PAH, emphasizing the early impact of COPD on vascular structure. The thickening and expansion of the smooth muscle layer in these vessels are likely driven by the proliferation of smooth muscle cells. IHC analysis of ECM proteins in lung samples from COPD patients indicates that abundant elastin can be detected in the early stages of COPD, and the abundance of collagen correlates with the thickness of the smooth muscle layer, suggesting that ECM protein deposition plays a significant role in COPD‐induced pulmonary vascular remodeling [22].

Oxidative stress is involved in COPD‐induced pulmonary vascular remodeling. ROS are important regulators of vascular tone and function, and NOX4 is the most abundant NOX isoform identified in endothelial and smooth muscle cells. It is a major contributor to increased ROS production in pulmonary artery smooth muscle cells [23], and its expression is associated with pulmonary vascular remodeling, vasoconstriction, and enhanced cell proliferation in the vascular wall; meanwhile, NOX4 is an effective regulator of ROS levels in pulmonary artery endothelial cells. The main transcriptional regulator of the hypoxic response is HIF. Overexpression of HIF‐1α increases ROS levels, while downregulation of NOX4 attenuates this effect, highlighting NOX4 as a downstream target of HIF‐1α that helps to elevate ROS levels under normoxic conditions [24]. Pulmonary vascular remodeling in COPD patients is associated with differential expressions of the HIF‐1α gene, which may be involved in the hypoxic pulmonary vascular remodeling process in COPD [25]. This study found that compared to the control group, pulmonary venules and arterioles in the COPD group exhibited high expression of NOX4 and HIF‐1α in smooth muscle cells and endothelial cells, suggesting that NOX4 and HIF‐1α are key mediators of pulmonary microvascular remodeling in COPD.

VEGF is one of the key mediators of angiogenesis, endothelial cell survival, and vascular permeability, and is believed to play a role in the pathogenesis of COPD. Studies have shown that high expression of VEGF is observed in the distal pulmonary arteries' smooth muscle and endothelial cells of COPD patients, and VEGF expression is correlated with increased serum HIF‐1α expression [26]. In this study, the serum VEGF concentrations were higher in the COPD group compared to the control group (*p* < 0.05), suggesting that VEGF may be involved in COPD‐induced pulmonary microvascular remodeling. Additionally, correlation analysis showed a positive correlation between serum VEGF and HIF‐1α protein expression in lung tissue homogenates (*r* = 0.45, *p* = 0.01), suggesting that HIF‐1α may regulate VEGF through certain mechanisms, contributing to COPD‐induced pulmonary microvascular remodeling.

Hypoxemia in COPD can regulate the activation of key transcription factors and signal transduction cascades, promoting inflammation and neutrophil infiltration, which leads to vascular remodeling [27]. Previous studies have also shown that macrophages are involved in the development of COPD, with NADPH oxidase playing a key role in this process [28]. Furthermore, HIF‐1α's downstream factor, VEGF, can serve as a chemokine for neutrophils [29] and is involved in the inflammatory mediator interactions that drive COPD pathogenesis, exacerbating the inflammation and creating a vicious cycle. Our study results indicate that peripheral blood neutrophil count in the COPD group was higher than in the control group (*p* < 0.001); it supports the presence of systemic inflammation even in mild‐to‐moderate COPD. Compared to the control group, COPD patients showed high expression of NOX4 and HIF‐1α in macrophages surrounding the pulmonary microvessels, suggesting that the NOX4‐HIF‐1α oxidative stress signaling pathway may contribute to COPD pulmonary microvascular remodeling by inducing macrophages around the pulmonary microvessels.

This study has several limitations that should be considered. First, the sample size was relatively small. Only patients with mild‐to‐moderate COPD without PAH were selected. In addition, all participants were recruited from a single center in China. Therefore, the findings of this study may not be generalizable to the broader population of patients with COPD. Second, while investigating the NOX4‐HIF‐1α signaling pathway, other potential contributing factors were not simultaneously evaluated, which may have influenced the results. Nonetheless, despite VEGF being regulated by various cytokines and molecular interactions, HIF‐1α‐driven transcriptional regulation remains the primary mechanism governing VEGF expression. Importantly, this study successfully established a significant correlation between VEGF and HIF‐1α expression, reinforcing its relevance in COPD‐related vascular remodeling. Third, the exclusion of PAH was based on echocardiography, which lacks the sensitivity of right‐heart catheterization.

In summary, this study concludes that pulmonary microvascular remodeling in COPD patients is characterized by several structural changes, including smooth muscle thickening, an increase in smooth muscle area, endothelial alterations, and ECM deposition. Notably, these vascular modifications occur even in the absence of PAH, emphasizing that COPD‐induced remodeling begins early in disease progression. The findings indicate that smooth muscle cell proliferation plays a key role in driving these changes and may contribute to the thickening and expansion of vascular structures. This underscores the significance of early vascular alterations in COPD and highlights the potential mechanisms underlying progressive vascular dysfunction in these patients. However, due to the cross‐sectional and observational nature of our study, causality between NOX4–HIF‐1α activation and microvascular remodeling cannot be established and requires further mechanistic investigation.

## Author Contributions

Guo Xiaotong and Xin Hongxia were responsible for conceptualization, supervision, funding acquisition and writing (review/editing). Chen Juan contributed to formulating the research questions, design and drafting the submitted manuscript. Fan Yuchun, Ji Ting, Cao Xia, and Jiang Haifeng assisted in data analysis and performed statistical analysis. All authors have read and agreed to the published version of the manuscript.

## Funding

This study was supported by the National Natural Science Foundation of China (No. 82460020), Natural Science Foundation of Ningxia Hui Autonomous Region (No. 2024AAC03574), and Ningxia Medical University Scientific Research Project (No. XM2023041).

## Ethics Statement

This study adhered to the ethical guidelines approved by the Ethics Committee of the General Hospital of Ningxia Medical University (KYLL‐2023‐0422). This study was conducted in accordance with the Declaration of Helsinki (as revised in 2024).

## Conflicts of Interest

The authors declare no conflicts of interest.

## Data Availability

The data that support the findings of this study are available from the corresponding author upon reasonable request.
